# Challenges and prospects of visual contactless physiological monitoring in clinical study

**DOI:** 10.1038/s41746-023-00973-x

**Published:** 2023-12-15

**Authors:** Bin Huang, Shen Hu, Zimeng Liu, Chun-Liang Lin, Junfeng Su, Changchen Zhao, Li Wang, Wenjin Wang

**Affiliations:** 1 AI Research Center, Hangzhou Innovation Institute, Beihang University, 99 Juhang Rd., Binjiang Dist., Hangzhou, Zhejiang China; 2 School of Automation Science and Electrical Engineering, Beihang University, Beijing, China; 3 Department of Obstetrics, The Second Affiliated Hospital of Zhejiang University School of Medicine, Hangzhou, Zhejiang China; 4 Department of Epidemiology, The Harvard T.H. Chan School of Public Health, Boston, MA USA; 5 College of Electrical Engineering and Computer Science, National Chung Hsing University, 145 Xingda Rd., South Dist., Taichung, Taiwan; 6 Department of General Intensive Care Unit, The Second Affiliated Hospital of Zhejiang University School of Medicine, Hangzhou, Zhejiang China; 7 Key Laboratory of Early Warning and Intervention of Multiple Organ Failure, China National Ministry of Education, Hangzhou, Zhejiang China; 8 Department of Rehabilitation Medicine, The First Affiliated Hospital of Zhejiang University School of Medicine, Hangzhou, Zhejiang China; 9 Department of Biomedical Engineering, Southern University of Science and Technology, 1088 Xueyuan Ave, Nanshan Dist., Shenzhen, Guangdong China

**Keywords:** Health services, Public health, Bioinformatics, Machine learning

## Abstract

The monitoring of physiological parameters is a crucial topic in promoting human health and an indispensable approach for assessing physiological status and diagnosing diseases. Particularly, it holds significant value for patients who require long-term monitoring or with underlying cardiovascular disease. To this end, Visual Contactless Physiological Monitoring (VCPM) is capable of using videos recorded by a consumer camera to monitor blood volume pulse (BVP) signal, heart rate (HR), respiratory rate (RR), oxygen saturation (SpO_2_) and blood pressure (BP). Recently, deep learning-based pipelines have attracted numerous scholars and achieved unprecedented development. Although VCPM is still an emerging digital medical technology and presents many challenges and opportunities, it has the potential to revolutionize clinical medicine, digital health, telemedicine as well as other areas. The VCPM technology presents a viable solution that can be integrated into these systems for measuring vital parameters during video consultation, owing to its merits of contactless measurement, cost-effectiveness, user-friendly passive monitoring and the sole requirement of an off-the-shelf camera. In fact, the studies of VCPM technologies have been rocketing recently, particularly AI-based approaches, but few are employed in clinical settings. Here we provide a comprehensive overview of the applications, challenges, and prospects of VCPM from the perspective of clinical settings and AI technologies for the first time. The thorough exploration and analysis of clinical scenarios will provide profound guidance for the research and development of VCPM technologies in clinical settings.

## Introduction

Visual Contactless Physiological Monitoring (VCPM) is an emerging technology that can measure the vital signs based on videos. It has been proven that VCPM is highly effective in monitoring blood volume pulse (BVP) signal, heart rate (HR), respiratory rate (RR), oxygen saturation (SpO_2_), and blood pressure (BP)^1–4^. More significantly, VCPM’s contactless characteristic offers clinical benefits such as user-friendliness, full automation, long-term monitoring, zero skin damage, improved clinical workflow efficiency and the greatly reduced risk of cross-infection. Particularly, VCPM can also play a critical role in combating cardiovascular diseases (CVDs) and offer full-cycle personal health management^5,6^.

**Fig. 1:**
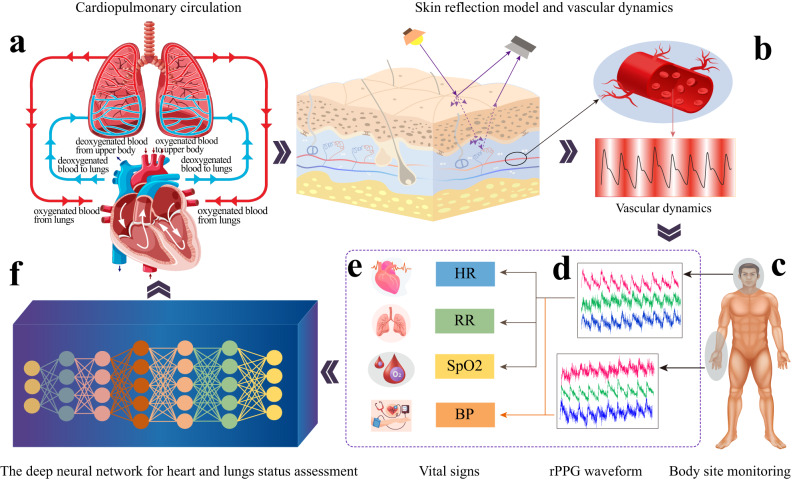
An overview of physiological principle of VCPM technologies of multiple physiological parameters monitoring. **a** A schematic representation of the cardiopulmonary circulation system. Due to the interaction of oxygen between the heart and lungs, respiratory rate information is implicitly reflected in hemodynamics. **b** The skin reflection model of the blood volume pulse (BVP) signal monitoring and the hemodynamics varying with the heartbeat. **c** Different body sites employed to extract PPG signals. **d** PPG signals from various body sites with RGB channels. **e** The vital signs derived from PPG waveforms. **f** The AI model for cardiopulmonary status assessment, and disease diagnosis. Subgraphs (**a**–**c** and **e**) are designed by Freepik.

As illustrated in Fig. 1, the basic physiological principles of VCPM are established on the cardiopulmonary and circulatory systems. As shown in Fig. 1a, the cardiopulmonary system facilitates the transportation of blood between the heart and lungs, whereas the blood moves from the aorta through the systemic arteries. In blood circulation theory, blood is ejected out of the heart and propagates along the arterial tree, and the BVP waveform takes on typical morphological components corresponding to landmark events (e.g., the contraction of left ventricle and the dicrotic notch) in the cardiac cycle^7^. Since blood flow is regulated by cardiac and respiratory interactions, it is theoretically possible to extract various physiological parameters through the analysis of a photoplethysmography (PPG) signal^7^.

Figure 1 illustrates remote PPG (rPPG) technology, approaches of physiological parameter measurement, and the solution of cardiopulmonary status assessment, which is broadly defined as VCPM technologies in this paper. As shown in Fig. 1b, owing to the fact that BVP waveform can be detected by the camera, rPPG technology is capable of extracting PPG signals from videos of the skin. Furthermore, PPG signals are employed to infer HR, RR, SpO_2_ and BP (Fig. 1c–e). SpO_2_ monitoring requires the measurement of at least two PPG wavelengths for SpO_2_ calibration. Additionally, BP can be measured by multi-site pulse transit time (PTT) (inferred from PPG waveforms from two distinct body sites), multi-wavelength PTT (from different skin layers), and morphological features. Moreover, in Fig. 1f, the vital signs can be employed to assess the wellness of cardiopulmonary system.

In 2000, Wu et al. proposed the first prototype of PPG imaging that uses an NIR light and black/white camera^8^. In 2007, researchers discovered that consumer RGB cameras can detect PPG waveforms in ambient light.^9,10^. After a decade, various camera-based rPPG algorithms, which were developed based on conventional computer vision and signal processing technology, have made a vigorous progress. Classic and popular algorithms include but are not limited to: CHROM^11^, PBV^12^, POS^13^, S^2^R^14^. In 2017, which can be called “the first year of deep learning for rPPG technology", scholars from University of Oxford and Taipei University of Science and Technology respectively presented their research achievements on newborn and adult subjects at the International Conference on Automatic Face and Gesture Recognition and the International Joint Conference on Biometric Recognition^15,16^. From 2021 to 2023, a variety of VCPM algorithms have emerged for the continuous monitoring of premature infants, babies, ICU patients, elderly people, etc^17–20^. Meanwhile, the number of the studies based on AI technologies with healthy subjects/laboratory environments has increased exponentially. Furthermore, PPG waveforms, derived from videos^20–22^, can be employed to infer HR^23,24^, SpO_2_^25,26^, RR^18,27^, BP^28,29^ and disease analysis^30–32^.

The COVID-19 pandemic over the past three years (2020–2022) has expedited the revolution of digital medicine^33–37^. The utilization of telemedicine systems experienced an exponential growth in numerous countries in the Organization for Economic Co-operation and Development (OECD) throughout 2020^38^. Compared to 2019, the number of Medicare fee-for-service beneficiary telehealth visits increased 63-fold in 2020, reaching nearly 52.7 million in the United States^39^. Similarly, in Germany, there were almost 1.4 million video consultations conducted during the first half of 2020^40^. In the second quarter of 2020 alone, patients consulted with doctors or psychotherapists via video almost 1.2 million times^40^.

Most importantly, COVID-19 has changed the context of digital medicine, and promoted the development of telemedicine and Primary Health Care (PHC) system^34,35,37,38,41^. Governments paid increasing attention to digital medicine and telemedicine systems, and patients gradually accepted this treatment approach^37,38,42^. During the COVID-19 outbreak, we have noticed that many countries and regions were suffering from a shortage of essential vital-sign monitoring equipment, particularly blood oxygen level monitors. Given that blood saturation is a critical biomarker that can be utilized to infer the likelihood of being infected with the COVID-19 disease, patients under home quarantine can judge whether they are developing lung infections or experiencing severe illness by these parameters^35,43–45^. Because of the utilization of off-the-shelf devices such as webcams or smartphone cameras for measuring vital signals, VCPM technologies can potentially solve the aforementioned challenges of medical equipment shortages. Based on these factors, VCPM technologies offer a natural and cost-effective approach to establishing digital medicine or PHC systems.

VCPM technologies based on deep learning have made tremendous progress in recent years, but the majority of these studies are limited to laboratory settings or healthy subjects. To apply these technologies to clinical medicine, there is a large space of improvement. Therefore, the motivation of this review paper is to re-examine the application of VCPM technologies in clinical healthcare monitoring, summarize the encountered challenges and issues, and enhance the fundamental theory of VCPM in clinical settings. Moreover, the prospect of developing a VCPM algorithm based on the state-of-the-art (SOTA) artificial intelligence technologies is depicted. Overall, this review offers the guidelines for the future development of VCPM algorithms toward clinical-grade applications.

The rest of the paper is structured as four sections. First, the search results of existing relevant works and study characteristics will be elaborated. Then, we will discuss the revolution of digital medicine, the merits of VCPM technology and the necessity of clinical settings in general. Next, the main challenges in clinical study will be illustrated in detail. Finally, the future directions and prospects of VCPM will be presented at length.

## Results

**Fig. 2:**
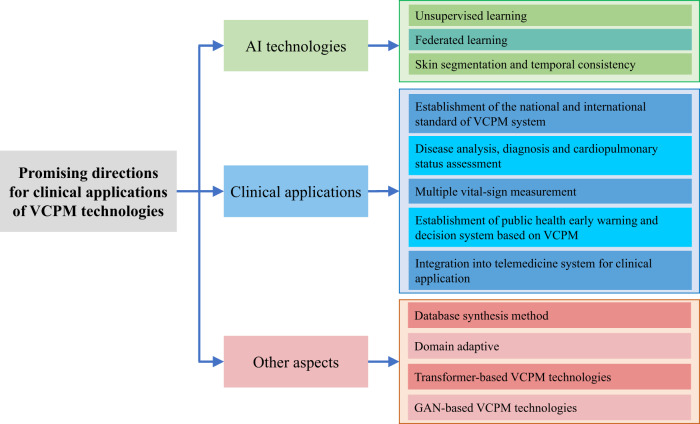
The pipeline of the future direction topics.

In this section, the future directions of VCPM will be summarized from three perspectives: the adoption of SOTA deep learning technologies and the breakthrough in the current limitations and clinical application challenges. The framework of this section is organized as Fig. 2. The establishment of a national and international standard of VCPM system is of top importance, and other parts can be divided into AI technology, clinical application and other aspects.

### Unsupervised learning

The unsupervised learning technique can be employed to establish AI models for the VCPM task without relying on ground truth vital signs during the training stage. Furthermore, unsupervised learning methods are typically more robust to noise and variations in data, making them ideal for real-world applications. In fact, not only the vital signs hidden in the skin video are weak, but the spatio-temporal features are intertwined^46^. Hence, it is a significant challenge to explicitly design neural network structures or loss functions to effectively decouple these spatio-temporal vital-sign features. Nevertheless, it is feasible to construct a reasonable strategy of unsupervised learning that enables the model to learn on its own and disentangle the intertwined features. For instance, recent research has demonstrated that unsupervised learning technologies are capable of extracting rPPG signals from unlabeled video data^47–53^. Moreover, the performance of those algorithms^48–51,54^ is comparable to or even better than that of supervised approaches.

### Federated learning

Federated Learning (FL) is a distributed machine learning paradigm or framework, and it is proposed to solve the *data island* problem of privacy protection. FL is capable of joint modeling without sharing participants’ data. The training dataset is stored in the local storage of participants, ensuring user privacy and complying with data usage standards^55,56^. In addition, FL technologies have arguably become the most widely used privacy preservation technique in AI-based medical applications^56–62^. Overall, FL technology is a promising solution to privacy protection, which can promote the R&D of VCPM for multi-centric clinical application studies. For instance, Liu et al. firstly developed a mobile FL camera-based PPG signal monitoring system with non-clinical public databases and showed that it can perform competitively with traditional state-of-the-art supervised learning methods^63^.

### Skin segmentation and temporal consistency

Due to the common occurrence of face occlusion and lateral face orientation in clinical settings, skin segmentation is a suitable solution that can effectively alleviate these unfavorable conditions. ROI extraction or skin segmentation is a critical preprocessing step for the VCPM task as only the skin surface can offer information of blood volume changes. In the earlier studies, various facial landmark detectors^64–66^ have been utilized to locate the ROI^67^. Nevertheless, it proves that these methods are ineffective in scenarios involving head movement or face occlusion, etc^17,68,69^. Additionally, Ouzar et al. demonstrated that face detectors^64–66^ might fail to detect ROI in the MMSE-HR dataset^70^, whereas this issue can be resolved by adopting a face segmentation algorithm^69,71^.

**Fig. 3:**
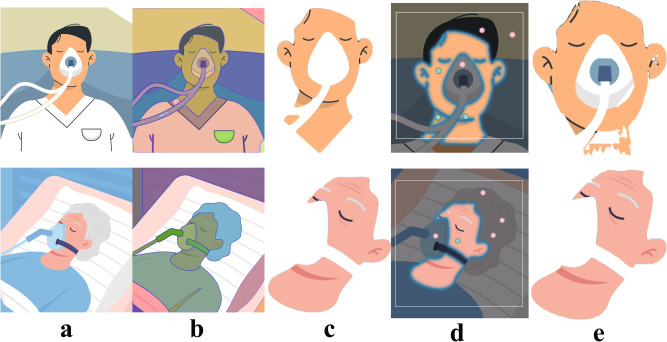
The skin segmentation results of SAM online demo on clinical scenarios. **a** The facial region of our ICU patients' recording image. **b** Full image automatic segmentation. **c** The results of automatic segmentation solution. **d** The interactive manual segmentation process. The rectangle box denotes the selected region, and dots represent the areas to be removed or retained. **e** The results of interactive segmentation. The source images are designed by Freepik.

It is important to accurately segment the skin ROI to extract vital signs effectively. Furthermore, skin segmentation has the ability to reduce noise and variation in original data, thereby improving the accuracy of VCPM algorithms and enabling vital signs extraction even under non-ideal conditions. In particular, skin segmentation is greatly crucial in clinical settings where the occlusion of the face of ICU patients is more prevalent. As shown in Fig. 3a, in the real-world scenario in ICU, face occlusion not only introduces additional noise, but also may cause failure in locating the patient’s face ROI if existing feature point detection algorithms are used. Therefore, by utilizing the SOTA semantic segmentation algorithms, we can obtain skin segmentation input at the pixel level, effectively mitigating interference from non-skin regions and enhancing the accuracy and robustness of the VCPM algorithm. The skin segmentation results, generated by the segment anything model (SAM) online demo (https://segment-anything.com/demo), on simulated clinical dataset are presented in Fig. 3.

Despite the introduction of segmentation algorithms by current pioneering researchers, the temporal consistency of continuous frames across video has not been taken into consideration^22,69^. Unlike single-image segmentation, temporal consistency is a critical metric that can significantly improve the performance of VCPM algorithms. Temporal consistency guarantees that the segmentation of each frame remains consistent with that of previous frames, which is crucial for accurate tracking of skin regions in videos over time. If the temporal consistency of the skin segmentation approach is suboptimal, the segmentation algorithm may introduce extra noise, ultimately leading to a decline in the performance of the VCPM algorithm. The AI-based optical flow is a potential research direction that can improve temporal consistency of video skin segmentation.

In 2023, various revolutionary segmentation tools^72,73^ were published, which provided great prospects for further improvement of VCPM algorithm performance. In Fig. 3, the results of facial skin segmentation using SAM^72^ in ICU patients under various complicated clinical conditions are presented. The segmentation results demonstrate that the background area is completely eliminated and the skin area is well preserved at a pixel-level precision. Thus, with the aid of advanced segmentation tools, the clinical VCPM algorithm can be trained with less background noise and more effective data, thereby increasing the feasibility of practical application.

### Establishment of the national and international standard of the VCPM system

VCPM technology is an accessible, comfortable, and convenient approach for physiological monitoring. To prevent the potential abuse or misuse of VCPM, it is essential to establish national and international standards and guidelines for its use in digital medicine. In terms of algorithm performance and data security, the standards should at least encompass the following aspects. Additionally, the recommended settings for the clinical application of VCPM technologies are listed in Table 1.

- **Video capture software and hardware settings**. The coded format of recording video is a crucial parameter for VCPM algorithm to extract vital signs. If the compression ratio of the collected video is too high, it may result in the loss of weak physiological signals implied in the video. The vital signs are time-domain information, therefore a stable and consistent sampling rate of the video is required. In addition, the resolution of the video is a crucial factor that ensures video quality and minimizes white noise. Hence, it is imperative to develop specialized video recording software to configure camera settings that can ensure the optimal performance of the VCPM algorithm.
- **Standard operating procedure (SOP)**. The SOP includes the lowest ambient light intensity, allowable subject motion magnitude, the shortest video duration and other considerations.
- **Data privacy protection**. Due to the fact that video data commonly cover both facial information and vital signs of subjects, protecting privacy is a crucial issue and a primary requirement. Formulate the criteria for video data accessibility based on the purposes and occupational categories. The related occupations include public individuals, physicians, researchers, pertinent government staffs and policy makers.

**Table 1:** Recommended settings for the clinical application of VCPM technologies.

Clinical settings / Parameters	Recommendation and announcement
Subjects’ state	Static or slight motion during measurement.
Illumination	If using RGB camera, illumination greater than 50 lux^186^, or normal ambient illumination and non-dark.
Camera selection	The embedded processing chips in some cameras may eliminate or weaken BVP signals. We have validated that Logitech C920/C922, HIKVISION DS-U102D/DS-65DC0403 and Intel RealSense D455 can be used to detect BVP signals.
Video resolution	Not lower than 640 × 480 px; recommend setting to 1080 × 720 px.
Video sampling rate	30 fps, the common used and supported by consumer camera
Video compression ratio	Generally, the lower the video compression rate, the easier it is used to observe BVP signals hidden in skin videos.
Measurement distance	According to the focal length of the camera, ensure the image of the skin region is clear, and the skin area is greater than 128 × 128 px (The input image size of AI-based methods is usually set as 128 × 128 px).
Measurement duration	HR: 5–10 s; RR: 15–30 s; SpO_2_: 2–10 s; BP: 5–20 s.
Measurement of BP and SpO_2_	Comprehensive and systematic verification is required to verify the feasibility in clinical practice. The tested BP and SpO_2_ database needs cover the range of 40–240 mmHg and 70–100% respectively.

**Table 2:** A summary table of clinical studies based on VCPM algorithm (The deadline of search is May 22, 2023).

First Author	Country	AI	Year	Subject (*n*)	Distance (cm)	Data length	Method and clinical condition	Vital-sign: ME ± SD (HR:bpm; RR:brpm)
Liu^147^	China	✓	2023	405	50–150	Each 10 min	Unsupervised learning; ICU patients; 67.8 Y/O. (mean); RGB; Chest ROI; 1440 × 1080/1080 × 720 px, 30fps	RR: 2.8 ± 3.0
Barde^156^	USA	×	2023	60	28–44	—	Atrial fibrillation patients; 67 ± 10 Y/O.; ≥50, 100, or 200 lux; Smartphone (Samsung S10 or S3), 30 fps	HR:0.05 ± 1.4
Batbayar^142^	Japan and Mongolia	×	2022	301	40	—	154 COVID-19 patients and 147 healthy individuals; Thermal (9fps, 80 × 60 px) and RGB camera (30 fps, 424 × 240 px); Lie in bed	HR:-0.2 ± 2.3; RR:0.0 ± 1.6
Svoboda^157^	Germany	—	2022	42	150	—	3D camera system containing RGB and NIR camera (950 nm); Newborn infants; 39.6 ± 1.3 weeks; 330.4 ± 13.9 lux	HR-3D:8.6 ± 4.4; 7D2HR-2D:3.0 ± 3.4
Allado^158^	France	—	2022	963	70–100	Each 60 s	Patients; 56.6 ± 16; Uncompressed video; BMI concordance; Fitzpatrick skin color scale concordance	HR:-0.1 ± 4.1
Allado^159^	France	—	2022	924	70–100	Each 60 s	Ditto	RR:-0.7 ± 3.5
Sahoo^127^	India	✓	2022	19	—	19 * 10 mins	An end-to-end DL model integrates a non-learning based approach; NICU; 25 fps, 1920 × 1080 px; NIR and RGB	HR: 0.8 ± 3.5
Zeng^160^	China	×	2022	10	focal length	—	Living-skin detection; Critically-ill premature infants; 160 × 90, 20 fps; RAW format video	HR:MAE = 5.68; RR:MAE = 10.82
Jorge^18^	UK	✓	2022	15	—	233.5 h	Post-operative ICU patients; 62.2 ± 12.4 Y/O.; Uncompressed 8-bit data (850 nm); 1024 × 768 px, 100 fps	HR:MAE = 2.5; RR:MAE = 2.4
Wang^161^	China	×	2022	10	—	each 10 min	ROI optimization using living-skin and respiratory maps; ICU Patients; CCTV camera in ceiling; 1920 × 1080 px, 25 fps	HR:1.7^△^ ± 6.0; RR:1.6^△^ ± 2.9
Ottaviani^162^	Italy	×	2022	12	>16	—	Extract 3D displacements of chest depth images; Preterm infants RR monitoring; RGBD, 90 fps, 1280 × 720 px	RR:0.02 ± 0.51
Hajj^163^	Canada	×	2022	4	—	—	NICU; Neonatal RR estimation with RGBD sensor (Intel SR300); Using automated ROI segmentation algorithm	RR:MAE =5.0
Pediaditis^149^	Greece	✓	2022	5	180	25–35 h	Using Eulerian magnification and 3D CNNs; Sleeping; 67 ± 10.8 Y/O; RGB and IR camera; 1080 × 720 px, 25 fps	RR:MAE = 2.29
Nagy^164^	Hungary	×	2022	10	80-150	240 h	ROI detection based on UNet++, but the principle method are not based on AI; NICU, GA:33.2 weeks (mean); Raw format, 500 × 500 px, 20 fps	RR: MAE = 1.09
Varma^165^	India	—	2022	158	122–152	Each 30 min	Adult patients’ HR and RR measurement in hospital; 18–80 Y/O; IDS UI3060 and CCTV camera	HR:−1.27 ± 5.6; 7D3RR: −0.3 ± 3.06
Huang^17^	China	✓	2021	257	50–100	9.6 h	In-hospitalized neonates (0–7 days); DL based method to extract PPG waveform and HR; 640 × 480 px, 30 fps	HR:−0.21 ± 5.32
Chen^166^	China	×	2021	9	26–36	4.21 h	Hospitalized newborns with motion artifacts; RGB camera; 640 × 480 px, 30fps; Mean GA: 36.6 weeks	HR-R:MAE = 3.4; HR-M:MAE = 4.3
Kyrollos^167^	Canada	×	2021	5	—	—	Video Magnification based method; NICU; RGBD camera	RR: MAE=3.5
Khanam^168^	Australia	×	2021	6	100–200	Each 10 min	Automatic ROI detection based on neural networks, but the principle method are not based on AI; NICU; Nikon D610 and D5300; 1920 × 1080 px, 30 fps	HR:0.44 ± 2.2; RR:0.71 ± 2.65
Laurie^169^	Australia	×	2021	7	—	—	patients with acute mental; 12 bit GigE camera; 648 × 488 px	RR:0.11 ± 0.86
Lyra^148^	Germany	✓	2021	26	—	—	Investigated skin temperature trend measurement and respiration-related chest movements; Using YOLO detector; ICU patient; IRT camera, 4fps; 382 × 288 px; Optical Flow	RR:−0.18 ± 2.8
Yu^170^	Germany	×	2020	20	180	25*20 min	Patient HR and HRV monitoring with NIR and RGB camera	HR: 0.0 ± 0.7
Villarroel^143^	UK	✓	2020	40	—	304.1 h	Patients undergoing haemodialysis treatment; 64.7 ± 15.3 Y/O; Uncompressed video format; 15 fps, 2448 × 2048 px	HR:0.0 ± 2.4; RR:0.2 ± 1.66
Malafaya^171^	Portugal	×	2020	3	—	15 min	Spectral and peak analysis; newborns in NICU; two cameras(Kinect V2 & Tesseract OCR engine); lateral	HR:RMSE = 6.93
Chen^172^	China	×	2020	5	25–36	5*3 min	EVM algorithm; infants in NICU(13-25 days); Fluke Tix580; right ahead; 640 × 480 px, 30fps	HR:7.4^ ±^ 12.5
Imano^173^	Denmark	×	2020	39	110	—	Estimate tidal volume (TV) and RR from chest depth image; Elderly people, 65-75 Y/O; still; RGBD, 30fps; right ahead	Tidal volume: :0.14 ± 0.03
Paul^174^	Germany	×	2020	19	70	240 min	Neonates; RFGB, IRT and NIR sensor at side of bed; short-time Fourier transform; 1920 × 1200/640 × 480 px, 30/25 fps	HR: ME=3.0
Negishi^175^	Japan	×	2020	28	100	—	Influenza patients(45 Y/O); RGB-thermal image sensors; right ahead; 640 × 480 px(visible); 320 × 240 px(thermal)	RMSE:HR:5.93 ± 5.85; RR&BT:1.73 ± 1.68
Villarroel^68^	UK	✓	2019	30	—	426.6 h	Preterm infants in NICU; GA:30.7 weeks; ECG sensor; above; 1620 × 1236 px	HR:0.3 ± 4.39; 7D3RR:−1.1 ± 5.66
Chaichulee^145^	UK	✓	2019	15	—	226.4 h	Preterm infants; above; 1620 × 1236 px; skin segmentation	ROI accuracy:94.5%
Rehouma^141^	Canada	×	2019	2	85 & 160	—	Children in PICU, 0-18 Y/O; still; two depth cameras; 45^∘^; 512 × 424 px	RR:0.8 ± 0.41(patient1); 0.7 ± 0.56(patient2)
Slapnicar^176^	Slovenia	×	2019	22	—	—	POS algorithm; adult patient; still; right ahead	HR:MAE = 7.92
Antognoli^177^	Italy	×	2019	40	50	—	Neonates; GA:35 ± 2 weeks; still; a uniform light source; above; 1280 × 720 px	RMSE: 6.8(HR), 2.1(RR)
Rasche^178^	Germany	×	2019	70	60–100	—	OPP; adult patients(70.3 ± 11.4 Y/O); fluorescent illumination and ambient light; right ahead; 420 × 720 px	—
Antognoli^179^	Italy	×	2018	7	50	—	Neonates; GA:32 ± 4 weeks; still; above; 1280 × 720 px, 30fps	HR:12.2,RR:7.6(RMSE)
Trumpp^180^	Germany	×	2018	41	50–100	—	Intraoperative patients(65.2 ± 12 Y/O); RGB and NIR cameras; surgical light; over head; 320 × 420 px	HR median: 95.6%(G),76.2%(NIR)
Chaichulee^146^	UK	✓	2018	20	—	110 h	Using MatConvNet to detect the head, torso and diaper; NICU; GA:≤37 weeks; still; 1620 × 1236 px, 20 fps	HR:2.4 ± 4.1
Cobos^181^	Spain	×	2018	—	50	—	Neonates; GA:25-40 weeks; natural/artificial light and skylights; above; 1920 × 1080/1280 × 720 px	HR:-1.5 ± 4.21, RR:0.6 ± 4.97
Blanik^182^	Germany	×	2016	10	50–100	Each 10 min	Neonates, 8-41 days; IR camera, infrared light emitting diode (LED) array (850 nm); above; 1920 × 1080 px, 25 fps	HR:−2 ± 9.69
Sikdar^183^	India	×	2015	14	—	—	Comparing the result of using RGB, RG, GB and RB channel; Infants(3–15 months) under Hammersmith examinations;	HR: –
Villarroel^144^	UK	×	2014	2	150	39.8 h	Neonates; GA:29 weeks; above; JAI AT-200CL digital 3CCD progressive scan, 1620 × 1236 px, 20 fps	HR:MAE = 2.83
Aarts^184^	Netherlands	×	2013	2	100	—	Neonates; 3 days–4 weeks; still; Blue Sensor NEOX, 150 lux.; 300 × 300 px, 30/15fps	HR:0.3 ± 2.68
Scalise^185^	Italy	×	2012	7	20	28 min	Neonates; GA:33 ± 2.5 weeks; large band light; right ahead; 640 × 480 px	HR:−0.9 ± 4.54

The databases are collected by the authors and are private.

*BMI* body mass index, *GA* Gestational ages, *Y/O* years old, *RGBD* RGB Depth camera, *IR* infrared, *NIR* Near infrared, *CCTV* closed circuit television, *EVM* Euler video magnification, *IRT* Infrared thermal imaging, *fps* Frames per second.

### Disease analysis, diagnosis and cardiopulmonary status assessment

With the aid of the AI technology boom, PPG, HR and heart rate variability can serve as biomarkers for disease analysis, diagnosis, and assessment of cardiopulmonary status^30–32,74–76^. Recently, numerous studies have demonstrated the high sensitivity and specificity of VCPM technologies in detecting atrial fibrillation^75,77–81^. Additionally, in literature^32^, a novel AI algorithm, which leverages PPG and ECG generated by PPG, has been successfully developed for CVD detection, including coronary artery disease, congestive heart failure, myocardial infarction (MI), and hypotension (HOTN). It can be seen that the utilization of VCPM technologies for monitoring vital signs and capturing their variations over days and weeks holds great potential in enabling early disease prediction and diagnosis^35,82^.

### Multiple vital-sign measurement

Despite the verification of VCPM technology in measuring HR, RR, SpO_2_, and BP, a multi-task AI model proficient in simultaneous detection of the four vital signs has not emerged. However, in clinical settings, it is imperative to concurrently monitor multiple vital signs to ensure comprehensive monitoring of the patient’s physiological status. In clinical patient monitoring, HR, RR, SpO_2_ and BP are the four essential parameters that comprehensively reflect the cardiopulmonary status of patients, and they are the fundamental indicators of the traditional multi-parameter patient monitors. If the VCPM framework can monitor multiple vital parameters simultaneously, it will be closer to the application of fully non-contact monitoring of patients in highly acute settings. Therefore, the study of multi-parametric measurement of AI models will make a crucial breakthrough in real-world clinical applications. Currently, the majority of researchers are primarily focused on developing contactless measurement algorithms for a single physiological parameter or two parameters with strong correlation, such as HR and RR. For instance, Villarroel and Jorge et al. have developed two AI models capable of monitoring HR and RR in clinical conditions^18,68^.

Fortunately, the VCPM technology has demonstrated the ability to simultaneously measure vital signs including HR, RR, SpO_2_ and BP^3,83–85^. Firstly, HR and RR can be derived from PPG signals^17,18,23,27,68,86–89^; Secondly, by analyzing two distinct PPG waveforms at the same measurement site, SpO_2_ can be computed^25,26,90–94^; Finally, utilizing two different PPG waveforms extracted from separate body sites enables the inference of both diastolic and systolic BP^1,2,4,95–99^. Therefore, the development of a large-scale and multi-task AI model, which has the capacity to simultaneously measure multiple physiological signals, holds significant clinical application potential and represents a promising direction for future research. In conclusion, it is greatly promising to establish a unified AI model incorporating multiple physiological parameters in clinical scenarios.

### Establishment of public health early warning and decision system based on VCPM

**Fig. 4:**
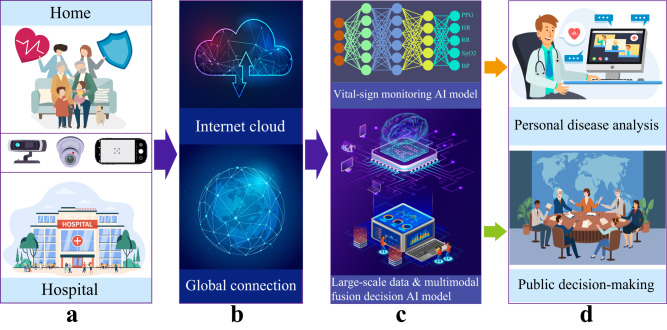
The VCPM-based telemedicine/telehealth system is employed to personalized disease diagnosis and public health management. **a** The two typical application scenarios. The VCPM relies solely on ubiquitous cameras to capture video data. **b** The internet infrastructure, including both wireless and wired networks, facilitates the transmission and storage of data across the globe. **c** The AI model of physiological monitoring based on individual video data, and the AI model for large-scale decision-making, incorporating multi-source information fusion based on global patient data. **d** The upper subgraph denotes the personal health care in a telemedicine system, while the lower subgraph depicts the decision-making of public health policies based on global patient information and horizontal relationships. The elements of sub-figures are designed by Freepik.

As illustrated in Fig. 4, the VCPM-based telemedicine system will not only be used for personal health monitoring and disease diagnosis, but also serves as an AI tool in response to public health issues, such as CVD in the elderly and the COVID-19 pandemic. Firstly, The vital signs of individuals measured by the VCPM-based telemedicine system can be utilized for personalized healthcare and disease diagnosis. Moreover, during an epidemic, the large-scale basic data of the public collected by the VCPM telemedicine system can be employed to establish a public health decision-making system, and offer crucial technical support for the government in formulating timely response strategies. For instance, numerous AI prediction models have been developed to predict the infected population and the mortality^100–102^.

### Integration into telemedicine system for clinical application

In telemedicine or telehealth system, video consultation is one of the must-have functions, which is a subjective approach of disease counseling. After the COVID-19 pandemic from 2020 to 2022, many telemedicine/telehealth systems have implemented this method to mitigate cross-infection risks between healthcare providers and patients during treatment for COVID-19 or other illnesses^103–106^. Therefore, VCPM can be easily integrated into those existing medical systems to support objective physiological information during video consultations with physicians. The implementation of this measure will further enhance the functionality of the telemedicine system and provide an exceptional user experience for those utilizing the remote system.

### Other recent research directions

#### Database synthesis method

It is a significantly challenging task to collect a large-scale and multi-centric database representing a range of environments, body movements, illumination conditions and physiological states. However, establishing a simulation video database integrated with physiological signals is a feasible solution for VCPM tasks^84,107,108^. For instance, in 2022, Daniel et al. released a synthetic database, named SCAMPS, which comprised 2,800 videos featuring synchronized cardiac and respiratory signals as well as facial action intensities^109^. Moreover, the synthetic data have the merits of noiselessness and precise synchronization. SCAMPS was successfully utilized to train AI models to develop the VCPM algorithms for healthy subjects^52,110^. Thus, developing a simulation database for clinical settings would be an invaluable future direction as it can mitigate the challenges of collecting extensive clinical data while safeguarding medical data privacy. It should be noted that the mathematical modeling of hemodynamics and oxygen saturation is a challenging task, which currently precludes the incorporation of oxygen and blood pressure information into simulation data.

#### Domain adaptive

Due to the bias (e.g., illumination, the bias of distinct clinical centers) between the training source and testing target domain, the generalization ability of deep learning-based methods should be introduced. To improve the generalization ability of rPPG models, Du et al. proposed a domain adaptive method that aligns intermediate domains and synthesizes target noise in the source domain to achieve superior noise reduction by reducing domain discrepancy^110^. We deem that the adaptive domain approach can be extended to effectively mitigate the disparity between laboratory scenario data and clinical data.

#### Transformer-based VCPM technologies

Transformer was first proposed in the field of Natural Language Processing (NLP)^111,112^. Then, another milestone event is the successful adaptation of Transformer for computer vision (CV) tasks, known as Vision Transformer (ViT)^113^. Nowadays, the Transformer module is renowned for achieving a unified architecture that utilizes self-attention mechanism to extract spatial (e.g., CV task) and temporal (e.g., NLP task) features simultaneously. Another characteristic of Transformer is its ability to handle various forms of input data fed into an embedded encoder. Overall, it is a promising direction to explore a Transformer-based VCPM framework which can extract spatial-temporal features and monitor multiple vital parameters in clinical settings. For instance, Wang et al. proposed a Transformer-based unsupervised learning model for remote HR measurement^53^.

#### GAN-based VCPM technologies

Generative adversarial network (GAN) is an unsupervised learning framework for estimating generative models via adversarial training^114^. GANs are widely utilized in the fields of data generation, data augmentation, style transfer, etc. Recently, GAN has been introduced to improve the performance and generalization of VCPM technologies^110,115–117^. Particularly, GAN is used to generate adversarial noise to improve the generalization ability of PPG signals’ prediction models^110,115^. Although some achievements have been made in studies on healthy subjects and laboratory settings, it also has significant value in clinical scenarios. Owing to the complex clinical scenarios and its distinction in different clinical centres, the generalization performance of AI-based VCPM algorithms might be degraded when applied to other clinical scenarios. Therefore, GAN-based VCPM technology is a potential approach to alleviating the generalization difficulty in multiple centres.

## Discussion

In the section, we will discuss the topics on (A) digital medicine revolution; (B) the merits of VCPM technologies; (C) The necessity of clinical settings; and (D) Main challenges in clinical study.

### (A) Digital medicine revolution

Key information

- Digital Medicine. Digital medicine is a comprehensive concept that encompasses the use of digital technologies, such as biotechnology, health technology, and biomedical engineering, to enhance healthcare delivery and improve patient outcomes through signal processing, artificial intelligence, machine learning, and big data analysis.
- Telehealth. Telehealth encompasses remote clinical healthcare, patient professional health education, as well as public health and healthcare administration. Usually, telehealth covers a significant proportion of digital health solutions.
- Telemedicine. Currently, there is no universally accepted definition of telemedicine. Generally, it is the utilization of telecommunications to remotely provide healthcare services, encompassing a wide range of applications such as video consultations, diagnosis and patient monitoring. It can be implemented through video conferencing, photo calling or special telemedicine software. Telemedicine is a component of the broader field of telehealth.
- Remote Patient Monitoring (RPM). RPM, a comprehensive technology solution, involves the utilization of sensors and other devices to remotely gather data on a patient’s health status, which can then be transmitted to healthcare providers for analysis and intervention. VCPM can be considered as one of the RPM techniques. This technology is applicable to monitor various conditions such as heart failure, diabetes, COVID-19^35^, and interstitial lung disease^33^.
- Self-monitoring and home-based monitoring. Self-monitoring refers to the utilization of digital tools and devices by patients to track their own health data, such as blood pressure monitors, glucose meters, and fitness trackers. This practice enables patients to proactively manage their health and detect potential diseases at an early stage. Home-based monitoring involves leveraging digital technologies to deliver healthcare services directly to patients in their residences.

The measurement physiological signals is a fundamental procedure for monitoring the body’s status, which is widely employed in clinical settings and daily health surveillance. VCPM, as a contactless measurement method, offers the benefits of user-friendly monitoring, passive monitoring and cost-effectiveness etc. Therefore, it has the significant potential for application in clinical settings or home-based monitoring, and is poised to revolutionize traditional medical devices, telemedicine, intelligent monitoring, and medicine industry.

**Fig. 5:**
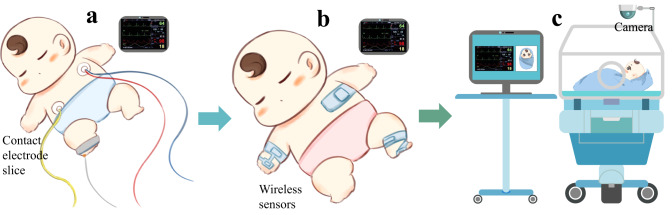
The development trend of the vital-sign monitoring of neonates or preterm infants. **a** The conventional contact monitoring approach with hard-wired devices and rigid sensors that adhere to neonatal skin. **b** The wireless, non-invasive soft biosensors employed to monitor physiological signals in NICU or pediatric ICU (PICU) settings, e.g., the research of literature^120^. **c** The video-based non-contact vital-sign monitoring solution utilized in the NICU, such as the study of Oxford University^68^.

As illustrated in Fig. 5, the current trend in the development of neonatal physiological signal monitoring instruments is shifting from wired contact to wireless contact monitoring^118–120^, and ultimately towards contactless measurement. In 2022, an AI-based contactless physiological monitoring algorithm was developed for post-operative patients in ICU settings. In the study, the VCPM algorithm measured the HR with a mean absolute error (MAE) of 2.5 beats/min in comparison to two reference HR sensors, and measured the RR with a MAE of 2.4 breaths/min against the reference value computed from the chest impedance pneumogram^18^.

### (B) The merits of VCPM technologies

**Fig. 6:**
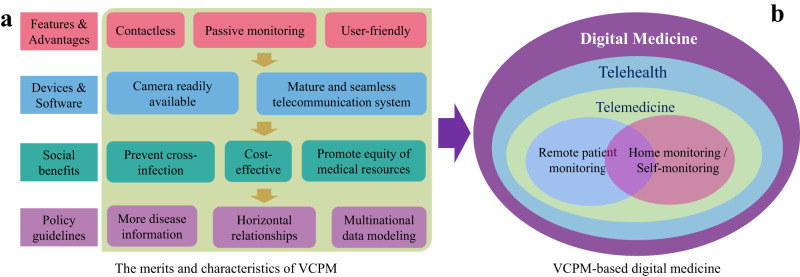
The digital medicine and telemedicine systems based on VCPM technologies. **a** The hierarchical advantages and characteristics of the VCPM methodology. **b** The relationships of concepts of digital medicine, telehealth, telemedicine, RPM, home-based monitoring or self-monitoring. The VCPM technology is a fundamental and suitable tool to support telemedicine, particularly in home-based monitoring.

Firstly, VCPM possesses a greater number of inherent and potential advantages. As illustrated in Fig. 6a, the approach of VCPM presents numerous merits, including contactless and non-invasive monitoring, passive measurement, user-friendliness, comfort and convenience, as well as suitability for long-term monitoring. Then, it leverages the ubiquitous devices and internet infrastructure at hand, including smartphones, webcams, and telecommunications systems. Therefore, the VCPM is a more natural method to establish telemedicine or home-based monitoring systems, and has the potential to yield significant economic and social benefits, including but not limited to preventing cross-infection among individuals/patients, reducing patients’ costs^121^, and promoting equitable distribution of medical resources.

### The merits of clinical applications

**Fig. 7:**
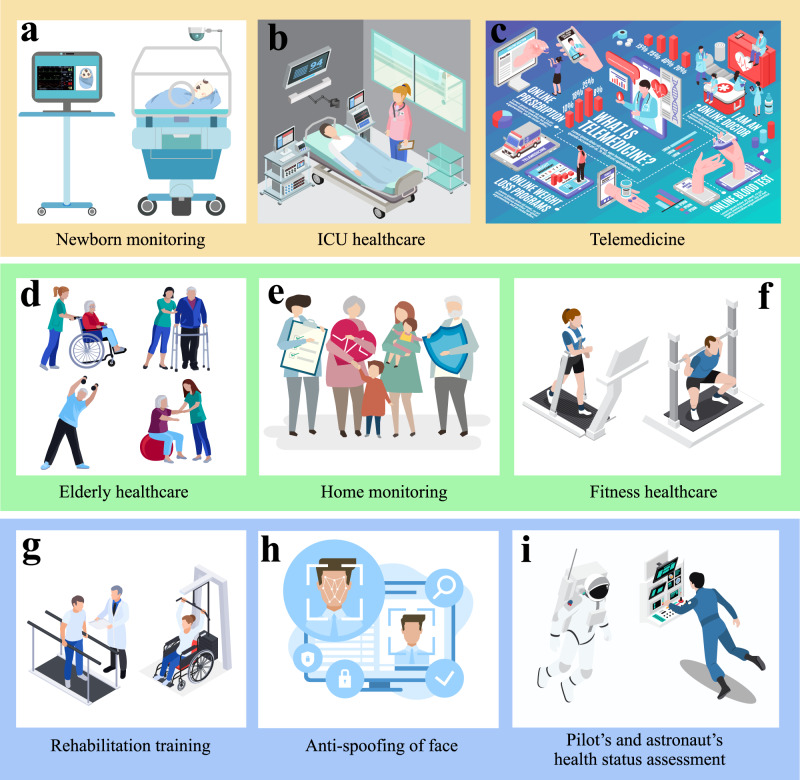
Application scenarios of VCPM. Sub-figure (**a**) is designed by our team, and (**b**–**i**) are designed by Freepik.

Due to the prevalence of camera devices and the convenient monitoring manner, the VCPM technologies have the potential to flexibly record public large-scale disease data and an individual’s physiological information. Meanwhile, big data and AI technologies have played a significant role in studying and recognizing brand-new diseases (e.g., predict infection rate and mortality) by utilizing large-scale vital signs from the public^41^. On one hand, VCPM establishes horizontal relationships between patients and providers, and makes multinational collaborations more feasible^41^. Moreover, as illustrated in Fig. 7, VCPM technologies have broad applications in various clinical scenarios, such as elderly care, newborn monitoring, ICU patient healthcare, rehabilitation training, and so on.

On the other hand, in terms of individuals, VCPM can be easily implemented on a large scale to track longitudinal changes, which are crucial medical indicators of their physiological status. Individuals undergo their own daily, weekly, and seasonal fluctuations in a variety of physiological parameters and activities. The earliest deviations from the norm can be detected only by establishing an individual’s baseline when they are healthy^44^. Therefore, owing to its flexible and passive manner, VCPM is significant for monitoring the physiological parameters of patients whether they are at home or in the hospital. For example, patients can transmit their skin video to the AI physiological signal monitoring system, and a physician can work remotely based on the vital signs. These advancements may encourage patients and their families to take greater ownership of their own healthcare. This system has the ability to reduce medical costs, decrease reliance on specialized equipment and physicians, promote equal distribution of medical resources, and improve the quality of healthcare services.

### The advantages of AI-based VCPM approaches

Firstly, it is an undisputed trend that utilizes SOTA AI technologies to develop VCPM algorithms. According to incomplete statistics, the vast majority of SOTA VCPM algorithms that were released from 2020 to 2023 are based on AI techniques. What’s more, the performance of deep learning methods has far exceeded that of signal processing methods. Secondly, AI technologies can not only be employed to develop the approaches of estimating multiple vital parameters, but also are capable of advancing post-process solutions, such as disease diagnosis with individual longitudinal analysis and other similar patient horizontal comparison^44^. In addition, the AI-based VCPM solution with the power of privacy protection presents a highly appealing option for clinical applications, such as utilizing AI-based approaches for protecting privacy^55,57,58^. Generally, the AI-based VCPM solution holds immense potential and offers significant advantages in the fields of clinical applications and digital medicine.

### The opportunities of VCPM-based digital medicine system

The VCPM will significantly expand the application range and scenarios of telemedicine and telehealth systems. A telemedicine/telehealth system typically encompasses the fundamental capabilities of biosignal measurement and video consultation. As illustrated in Fig. 6a, the VCPM approach utilizes existing infrastructures, such as webcams and the Internet, to establish a telemedicine/telehealth system without specialized medical equipment. Therefore, as shown in Fig. 6b, VCPM is particularly well-suited for establishing a telemedicine system that integrates remote patient monitoring, home-based monitoring, and video consultation simultaneously. It can be regarded as one of the essential underlying technologies for telemedicine systems, especially in supporting remote patient monitoring and home-based healthcare.

Ultimately, VCPM technology offers an unprecedented opportunity to self-health monitoring, PHC^41^ and telemedicine system due to the distinctive merits of VCPM, which include contactless operation, user-friendly interface, low cost and non-requirement for medical professionals. VCPM can be applied across the entire spectrum of prevention, diagnosis, and treatment. It is competent method to facilitate self-physiological signal monitoring and health status assessment in the stage of disease prevention. Furthermore, it can be integrated into PHC and telemedicine systems with fundamental physiological data for diagnosis. It serves as a tool to monitor the body’s physiological state, and can be applied widely as illustrated in Fig. 7.

### (C) The necessity of clinical settings

Firstly, multi-parameter monitoring is a necessary approach to maintaining the life and health of preterm / newborn infants. In 2020, World Health Organization reported that approximately 35% of all under-5 deaths occurred within the first week of birth^122^. For newborn or preterm infants, vital-sign monitoring is a fundamental clinical requirement because the fetal-to-neonatal transition after birth is a complex physiological process that affects all organ systems^123–126^. Moreover, it is also an indispensable procedure in the neonatal intensive care unit (NICU) environment. However, traditional contact-based methods are uncomfortable even harmful over the long-term contact of sensors. Thus, the visual contactless pipeline provides a notable competitive advantage in vital-sign monitoring by providing a convenient and contactless approach^17^. For instance, some pioneering studies have been conducted on hospitalized neonates based on deep learning^17,68,127,128^.

Furthermore, the majority of clinical patients require vital-sign monitoring, particularly those who are critically ill, have had surgery or suffered from CVD^129^ or hypertension. For patients who require long-term monitoring, traditional contact monitors have obvious clinical disadvantages. If the sensor probe is too tight, it can cause skin damage during extended use. Conversely, if the probe is too loose, it may easily detach due to the patient’s movement and necessitate professional reattachment. The primary unmet need being addressed by non-contact monitoring solutions is the mitigation of patient discomfort caused by contact or wearable monitoring technology^130^. For instance, wearable sensors are difficult to use in some patients with cognitive impairment (e.g., Alzheimer’s disease)^75^.

### (D) Main challenges in clinical study

Compared with the studies based on healthy subjects or laboratory scenarios, the clinical application of VCPM faces a multitude of unique challenges and the number of clinical studies is extremely limited. Therefore, the study of VCPM techniques is highly valuable in addressing digital healthcare challenges in real-world clinical scenarios^131^. Certainly, the following disadvantages of the VCPM technologies must be taken into consideration when applied in clinical settings: (1) Privacy protection; (2) Requiring substantial clinical validation; (3) Not suitable for dark environment unless using an infrared camera; (4) The performance susceptible to disturbance, such as head movement. In addition to the aforementioned issues, the primary obstacles that VCPM faces in clinical study are drawn out in this section.

### I. A shortage of public clinical database

The primary challenge lies in the absence of a publicly available database of clinical scenarios. To date, some pioneering studies have been conducted on clinical patients, but none of those data is available due to the patients’ privacy protection. Villarroel et al. conducted research on the application of VCPM algorithms in post-operative patients^18^ and preterm infants^68^ in the intensive care unit (ICU) respectively. However, those corresponding database are not publicly accessible. Moreover, there are only 15 ICU patients and 30 preterm infants recruited in literature^68^ and^18^ respectively. The limited amount of clinical data from a single center are insufficient to support further research and optimization of AI algorithms, as well as the clinical application of VCPM algorithms.

The scarcity of a clinical public database seriously impedes the algorithmic and application innovation in the VCPM research community. First, due to the unavailability of clinical data, the barriers to the clinical research of VCPM are increased. Thus, a significant proportion of scholars fail to carry out research smoothly. Next, there is no unified benchmark for comparing algorithms developed by various researchers. Last, it would hinder the healthy and sustainable development of the research community. Overall, it is imperative and opportune to establish a public database on its clinical scenarios.

The main deterrent to releasing clinical data results from safeguarding patients’ privacy^132,133^. Generally, the data recorder for VCPM includes the facial video of patients and multiple physiological information. Hence, providing access to the data for researchers in need while ensuring privacy presents a tricky issue. To this end, it is necessary to establish new standards for privacy and disclosure of clinical databases by collaborating with the government, academia, and medical community. These guidelines will revolutionize the development and application of AI technologies in digital medicine. From the perspective of technology, there are at least two potential solutions to achieve this objective: establish an AI-based privacy protection system or simulated database for clinical studies.

On the one hand, the primary concept behind federated learning systems is to construct machine learning models utilizing database that are distributed across multiple devices, while simultaneously preventing any potential data leakage^134,135^. Recently, federated learning have been widely applied in healthcare and clinical systems^57,58,136–138^, and just one VCPM study leverages federated learning at present^63^. On the other hand, simulation dataset leverages the concept of digital twins, utilizing both the original clinical video and corresponding physiological signal data to construct a simulation database. Thus, some researchers just need to access the simulation dataset to develop their AI algorithms, and then adopt the transfer learning to optimize the model trained on simulated data.

### II. Complex clinical scenarios

Due to the extremely weak vital signs hidden in facial videos, they are susceptible to interference from subjects’ status and surroundings. For instance, face occlusion and lateral face videos can weaken physiological signals, while head motion and illumination changes will enhance disturbances. Ultimately, these negative factors increase the challenges in developing a robust VCPM algorithm.

### Face occlusion and lateral face orientation

Oxygen therapy is commonly applied to ICU patients, but it will obscure parts of the face due to the presence of oxygen tubes and fixed coated fabric. Moreover, the oxygen tubes are situated in various regions of the face. Thus, it is a time-consuming and laborious task to segment them from each frame of the facial videos. In addition, unlike healthy subjects in laboratory settings, clinical patients can not be instructed to face the camera and are typically confined to their sickbeds with a lateral orientation. Moreover, to minimize background interference and maximize the retention of skin that contains physiological signal information, it is necessary to eliminate non-skin region as much as possible before we feed the skin regions into a VCPM algorithm. For videos of the healthy subjects’ face, a facial landmark tool is commonly utilized to extract face ROI, but the tool is not usually applicable to subjects with occluded or laterally oriented face^139,140^.

### Head motion and illumination changes

Furthermore, the current bottleneck of VCPM solution lies in their algorithmic performance, which fails to meet clinical measurement accuracy requirements when subjects experience head motion or surrounding illumination changes. The essential reason is that the amplitude of weak vital signs concealed in facial videos is significantly lower than the noise caused by head motion and illumination changes. There have been a few pioneering scholars attempting to tackle these hot-potato issues, yet much work remains for VCPM technologies to attain their full potential, particularly in medical field.

### III. Confidence evaluation of algorithms

Due to head movements or illumination changes, the performance of VCPM algorithms may become worse. Therefore, it is reasonable to introduce a confidence evaluation to assess results. The real-time presentation of the confidence coefficient indicates the level of confidence in the measured vital signs. The confidence level can be regarded as a metric of evaluating the algorithm’s adaptability to clinical scenarios. Furthermore, it will not only facilitate physicians in assessing patients’ conditions, but also provide guidance for further algorithmic improvement to researchers.

### IV. Pathological feasibility analysis

**Fig. 8:**
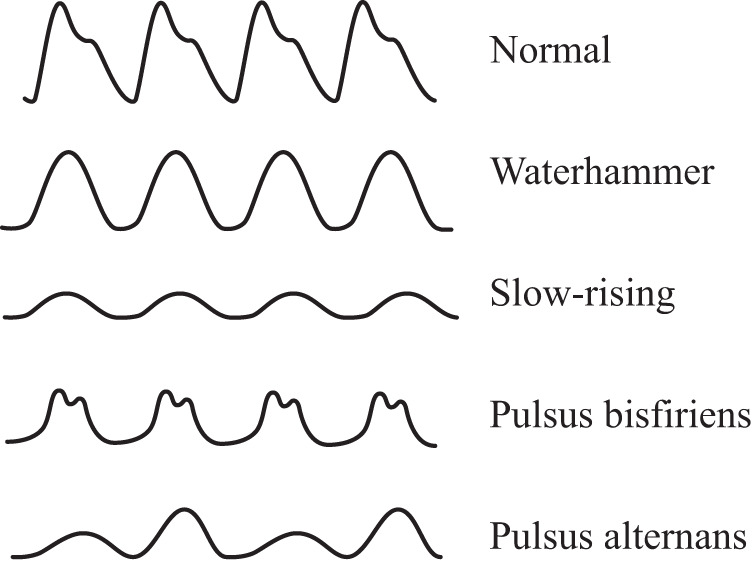
The distinct types of abnormal PPG waveforms.

In clinical practice, there is a high prevalence of hypertension and CVDs among the elderly population, resulting in various abnormal PPG signals. As shown in Fig. 8, the morphological characteristics of abnormal PPG signals are greatly different from that of normal PPG waveforms. As illustrated in Fig. 1, the fundamental principle of VCPM algorithm is based on the current normal PPG signal. Therefore, it is a tough task to develop a clinical VCPM algorithm that can adapt to abnormal PPG signals and further infer HR, RR, BP, and SpO_2_, which is also urgently needed validation in clinical studies.

However, there has been no study investigating the impact of abnormal pathological PPG signals on the performance of VCPM algorithms so far. All studies assume that subjects have normal PPG waveforms. Hence, comprehensive research guarantee that the effectiveness and robustness of VCPM algorithm is applicable to abnormal PPG signals in clinical situations, which is a challenging task and an innovative future direction to develop an effective VCPM pipeline. Furthermore, VCPM-based technologies can be developed for the diagnosis of CVDs.

Figure 8 displays five PPG waveforms, including four commonly seen abnormal PPG signals in clinical settings. Similarly, the abnormal PPG waveforms were detected in our clinical studies uses a finger-clip sensor. The waterhammer PPG is characterized by a sudden increase in the amplitude of the PPG signal, followed by a gradual decrease. The slow-rising PPG is identified by a prolonged rise time, which refers to the duration from the onset of the blood volume change to the peak of the signal. Specifically, the slow-rising PPG has a longer rise time compared with normal signals.

Pulsus bisferiens, meaning “beating twice", is a type of arterial pulse characterized by two distinct systolic peaks resulting from a rapid rise in blood pressure during systole, followed by a brief fall and then a second rise. This phenomenon is most commonly associated with aortic regurgitation, which stems from the incomplete closure of the aortic valve during diastole, leading to retrograde blood flow into the left ventricle, causing an increase in stroke volume consequently.

Pulsus alternans is a condition distinguished by alternating strength of the arterial pulse between beats due to variations in stroke volume. A decrease in stroke volume leads to weaker pulses, while an increase results in stronger ones. This phenomenon is most commonly related to the left ventricular dysfunction like that observed in heart failure.

## Methods

### Search results

**Fig. 9:**
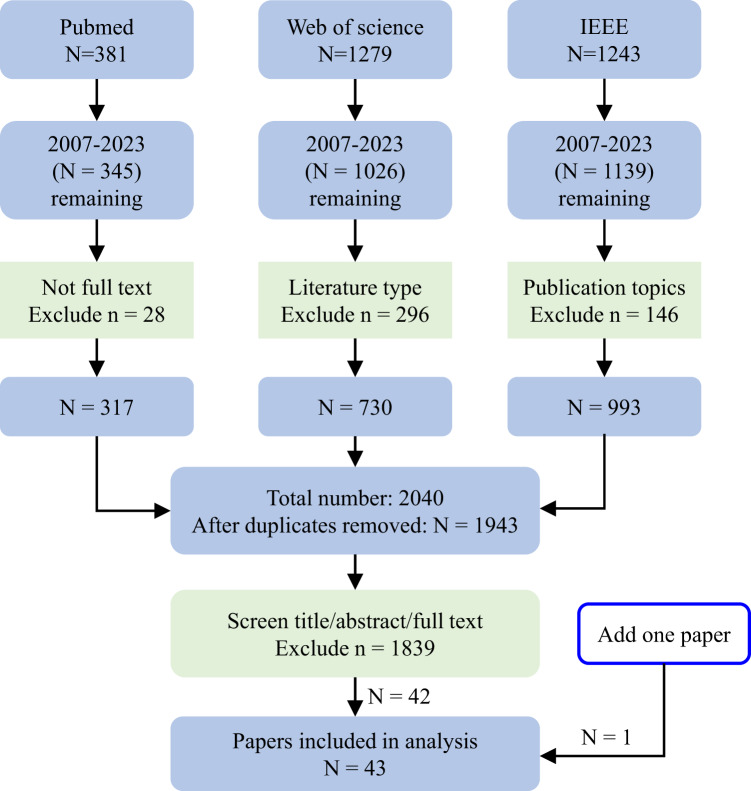
Flowchart for literature search and screening.

We retrieved a total of 381, 1279 and 1243 records from three databases (Pubmed, Web of Science (WOS), and IEEE) respectively (Fig. 9). Initially, we applied time filters due to the commencement of VCPM research in 2007, which resulted in a remaining total of 345, 1026, and 1139 records respectively. Subsequently, specific built-in filter tools of the three databases were employed: (1) Exclude 28 records with *not full text* filter in Pubmed; (2) After filtering out literature types that include review papers, unspecified material, books, abstracts only and letter, 730 papers remain in the search results of WOS; (3) Utilizing the filter of publication topics (patient monitoring, medical image processing, medical signal processing, cardiology, diseases, health care, biomedical optical imaging, telemedicine, medical signal detection or cardiovascular system) and 993 records remain in the search results of IEEE. Next, after eliminating the duplicates, there were 1943 items remaining. Finally, after screening by title, abstract or full text, studies conducted in laboratory settings or using radar sensors (e.g., MMW radar) were excluded. Only research papers related to clinical settings, digital medicine, telemedicine or healthcare were selected for final analysis, resulting in a total of 43 papers.

### Study characteristics

The 43 research papers are listed in reverse chronological order in Table 2. There were 24 papers in which studies were based on neonates or premature infants, the subjects of another 18 papers were adult patients, and the subjects of the last one included newborns and children^141^. Besides, Batbayar et al. developed a rapid preliminary COVID-19 screening system integrated with a stereo depth, an RGB and a thermal camera to measure RR, HR, and body temperature (BT) respectively^142^. The six studies (Villarroel et al., 2020, 2019, 2014; Chaichulee et al. 2019, 2018; Jorge et al., 2022)^18,68,143–146^ were from the same team at Oxford University. Among these studies, four papers focused on neonates while the remaining two were for adult patients.

In terms of the implemented algorithm, there were 29 research papers (29/43, 67.4%) that conducted classical methods, and only nine studies (10/43, 23.3%) implemented AI-based methods. Besides, the remaining four articles (4/43, 9.3%) did not explicitly state the used methods. Among the ten AI-based papers, five studies were from the team of Oxford University (UK)^18,68,143,145,146^, two from Beihang University (China)^17,147^, and the remaining three from RWTH Aachen University (Germany)^148^, Indian Institute of Technology Madras (India)^127^, Institute of Computer Science FORTH (Greece)^149^ respectively.

**Fig. 10:**
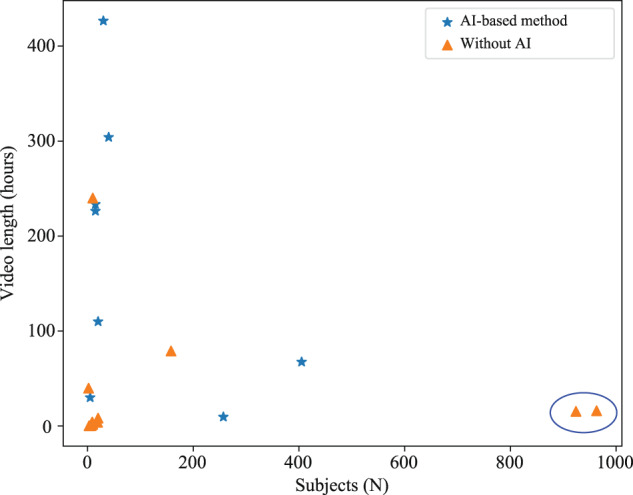
The relationship between the number of subjects and videos’ length.

Additionally, the relationship between the number of subjects and total videos’ length are presented in Fig. 10 based on the data resources shown in Table 2. Despite the 23 pairs of data may not be entirely statistically significant, Fig. 10 presents that the data scale (video total length or number of subject) of AI-based methods are commonly larger than non-AI approaches when excluding studies inside the blue ellipse from the same team. Generally, the performance of AI-based approaches is dependent on the scale of database, while classical methods only require fewer data samples. In fact, no matter AI-based approaches or classical ones, a large-scale and diverse database is indispensable to assess the comprehensive performance of VCPM approach toward complex real-world clinical settings.

In addition, although contactless studies in clinical scenarios have been emerging, some critical physiological indicators such as SpO_2_ and BP have not been researched so far. The research contrast of physiological parameters between laboratories and clinical scenarios is illustrated in Table 3. Depending on the selected clinical studies and the SOTA research trends of VCPM, our findings are summarized below:

- All clinical studies are based on their own database. The vast majority of these studies focus on the assessment of clinical applications, rather than dealing with real-world clinical scenarios, researching novel paradigm or analyzing clinicopathology.
- As shown in Tables 2 and 3, all clinical studies concentrate on the measurement of HR or RR. Even though AI-based research developed for SpO_2_ or BP measurement on healthy subjects has been growing explosively from 2021^90,96^, none of them are intended for monitoring SpO_2_ or BP in clinical settings.
- The VCPM algorithms, developed for clinical settings, are increasingly favored by researchers at present. In particular, the research on VCPM algorithms showed an exponential growth in 2022.
- There is a great gap between healthy/laboratory scenarios and clinical settings on the research of AI-based methods. A great many SOTA AI-based methods have been developed on healthy/laboratory settings^20^, but none of them has been applied in clinical settings.

**Table 3:** The quantitative contrast of physiological parameters between laboratories and clinical scenes.

Parameters	Healthy people / Laboratories	Patients / Clinical scenes (ME ± SD)
HR (bpm)	MAE = 0.6, RMSE = 1.83 (UBFC); MAE = 0.6, RMSE = 1.84 (PURE)^187^	0.05 ± 1.4^156^
	MAE = 0.16, RMSE = 0.6 (UBFC); MAE = 0.85, RMSE = 2.1 (MMSE-HR)^110^	−0.1 ± 4.1^158^
	RMSE = 0.765 (OBF)^188^	−0.2 ± 2.3^142^
RR (brpm)	MAE = 1.28, SD = 2.33^189^	MAE = 2.8, SD = 3.0^147^
	MAE = 1.62, SD = 2.1^23^	−0.7 ± 3.5^18,159^
	MAE = 1.76, RMSE = 2.6 (The mean values of the four modes)^190^	MAE = 2.4^18^
SpO_2_ (%)	MAE = 1.19, RMSE = 1.36^25^	×
	MAE = 0.88, RMSE = 1.22^93^	×
	MAE = 1.26^94^	×
BP (mmHg)	SBP: MAE = 2.4, RMSE = 4.2; DBP: MAE = 2.0, RMSE = 3.5^191^	×
	SBP: MAE = 8.7, SD = 9.9; DBP: MAE = 5.5, SD = 6.9^97^	×
	SBP: MAE = 9.1, SD = 8.2; DBP: MAE = 8.8, SD = 6.1^116^	×

All reference studies were published from 2022 to 2023.

**Phenomenon: the gap between the laboratory and clinical settings**

1. The studies of AI-based VCPM algorithm have been growing exponentially on healthy subjects/laboratory settings from 2021 to 2023. As shown in Table 3, the SOTA algorithms have demonstrated outstanding performance, but have rarely been generalized to clinical application.
2. The studies of VCPM algorithms have been soaring on patients/clinical settings from 2021 to 2023, yet only a limited number of studies have incorporated AI-based algorithms. In Table 1, only 6 papers (6/21, 28.6%) utilized AI technologies (2021–2023).
3. From the perspective of the novelty of approaches based on AI technologies, unsupervised learning^47,48,51^, Transformer^49,150–152^, GANs^115–117^, meta-learning^153^, and Graph Neural Networks^154,155^ have been developed for non-clinical scenarios. However, these technologies are rarely utilized for clinical settings.
4. As illustrated in Table 3, although the AI-based study about contactless SpO_2_ and BP estimation has become a hot topic in recent two years, all the studies concentrated on healthy people rather than the patients in the hospital.

**Reason: thinking and inference**

1. The SOTA AI-based VCPM algorithms developed in laboratory settings still face significant challenges in clinical application. Further verification is required to confirm the performance of these algorithms when generalized to clinical scenarios.
2. Because of the privacy protection of patients, there is still a shortage of large-scale and accessible clinical databases for the researchers of computer vision and AI. This seriously hinders the development and application of AI-based VCPM algorithms in the clinical environment.
3. Particularly, the performance of SpO_2_ and BP measurement algorithm in clinical settings is urgently need to be validated. It has crucial guiding significance for subsequent clinical studies of VCPM technologies. The performance evaluation of measuring SpO_2_ should cover the range of blood oxygen levels from 70% to 100%. To comprehensively evaluate the performance of the BP measurement algorithm, it is necessary to recruit sufficient hypertensive and hypotensive patients respectively.

**Prospect: possible solutions**

1. It will represent a significant milestone to establish a large-scale publicly accessible clinical database and usage standard for VCPM researchers. The database includes video, PPG, HR, RR, SpO_2_, and BP information. The greatest obstacle to achieving public access to the clinical database is privacy protection. Therefore, the exploration of a publicly available clinical database that protects privacy is one of the crucial future research directions.
2. The database will promote the study of VCPM algorithms in clinical settings, achieve a fair comparison of algorithm performance, and facilitate the sustainable development of the VCPM community.
3. Moreover, the database will attract plenty of excellent researchers in the field of computer vision and AI to join the clinical VCPM community. Ultimately, it will bridge the current great research gap between laboratory and clinical settings, and accelerate the clinical applications of VCPM technologies.

### Reporting summary

Further information on research design is available in the Nature Research Reporting Summary linked to this article.

### Supplementary information

Reporting Summary
